# Production of scopularide A in submerged culture with *Scopulariopsis brevicaulis*

**DOI:** 10.1186/1475-2859-13-89

**Published:** 2014-06-18

**Authors:** Anu Tamminen, Annemarie Kramer, Antje Labes, Marilyn G Wiebe

**Affiliations:** 1 VTT Technical Research Centre of Finland, P.O. Box 1000, FI-02044 VTT, Finland; 2 Kiel Centre for Marine Natural Products KiWiZ, GEOMAR Helmholtz Centre for Ocean Research, Kiel, Am Kiel-Kanal 44, Kiel 24106, Germany

**Keywords:** Marine fungi, *Scopulariopsis brevicaulis*, Scopularide A, Stirred tank bioreactor

## Abstract

**Background:**

Marine organisms produce many novel compounds with useful biological activity, but are currently underexploited. Considerable research has been invested in the study of compounds from marine bacteria, and several groups have now recognised that marine fungi also produce an interesting range of compounds. During product discovery, these compounds are often produced only in non-agitated culture conditions, which are unfortunately not well suited for scaling up. A marine isolate of *Scopulariopsis brevicaulis*, strain LF580, produces the cyclodepsipeptide scopularide A, which has previously only been produced in non-agitated cultivation.

**Results:**

*Scopulariopsis brevicaulis* LF580 produced scopularide A when grown in batch and fed-batch submerged cultures. Scopularide A was extracted primarily from the biomass, with approximately 7% being extractable from the culture supernatant. By increasing the biomass density of the cultivations, we were able to increase the volumetric production of the cultures, but it was important to avoid nitrogen limitation. Specific production also increased with increasing biomass density, leading to improvements in volumetric production up to 29-fold, compared with previous, non-agitated cultivations. Cell densities up to 36 g L^-1^ were achieved in 1 to 10 L bioreactors. Production of scopularide A was optimised in complex medium, but was also possible in a completely defined medium.

**Conclusions:**

Scopularide A production has been transferred from a non-agitated to a stirred tank bioreactor environment with an approximately 6-fold increase in specific and 29-fold increase in volumetric production. Production of scopularide A in stirred tank bioreactors demonstrates that marine fungal compounds can be suitable for scalable production, even with the native production organism.

## Background

Marine organisms produce many novel compounds with various biological activities [1,2]. Until recently, much of the research has focused on compounds from marine invertebrates, bacteria, cyanobacteria and dinoflagelates, but interesting compounds have also been isolated from marine fungi [3,4]. During product discovery, these compounds are generally identified as extracts from strains growing in non-agitated conditions, which are convenient for large scale product discovery, but not well suited for scaling up [5-7].

A strain of *Scopulariopsis brevicaulis* (LF580) which was isolated from a marine sponge was shown to produce an interesting cyclodepsipeptide, scopularide A [8]. Scopularide A consists of five amino acids (glycine, L-phenylalanine, L-alanine, L-valine and D-leucine) and hydroxymethyldecanoic acid. It inhibited the growth of both pancreatic and colon tumor cell lines [8] and may be of interest for further studies. Yu et al. [8] grew *S. brevicaulis* in complex medium (Yeast Malt Peptone, YMP, with 30 g L^-1^ NaCl) containing 10 g L^-1^ glucose and 3 g L^-1^ malt extract as the primary carbon sources, for 14 days to produce 7 mg L^-1^ scopularide A (approximately 0.02 mg L^-1^ h^-1^) in non-agitated cultivation conditions (static flasks). Neither the conditions of production nor the biosynthetic pathway were investigated.

*S. brevicaulis* (teleomorph *Microascus brevicaulis*, formerly *Penicillium brevicaule*) is commonly isolated from soil environments. Soil isolates have been used to volatilize arsenic and to methylate antimony [9], and to produce keratinase for degradation of feathers [10] or methioninase, an enzyme with potential medicinal application [11]. *S. brevicaulis* has been isolated as an opportunistic human pathogen, causing infections of skin or nails. Some strains have been isolated from high salt environments or shown to tolerate high concentrations of salt [12,13]. It has not often been reported from marine environments [14], nor have there been reports of scopularide production from other isolates, probably because they have not been assessed.

In this paper we demonstrate that scopularide A is produced by *S. brevicaulis* LF580 in stirred tank bioreactors (STRs) and describe the growth and scopularide A production of *Scopulariopsis brevicaulis* LF580 in STRs from 500 mL to 10 L scale.

## Results and discussion

### Scopularide A was produced in agitated conditions

Scopularides were produced in shaken flasks in the YMP medium described by [8], but production was variable: varying from 2 to 25 mg L^-1^ scopularide A. Shaken flasks contained relatively large volumes of culture (up to 50%) and were agitated under mild conditions, as a first step in the transition from static to agitated cultivation. Thus shaken flasks would have generally been oxygen limited, with variable hyphal morphology, both of which probably contributed to the high degree of variation observed in scopularide A production. However, the range observed indicated that scopularides could also be produced in STRs.

In STR, 29 ± 3 mg L^-1^ scopularide A (mean ± standard error of the mean (sem); ~2.4 mg [g biomass]^-1^) were produced after 90 h cultivation (Table 1). Both volumetric and specific scopularide A concentrations were improved, relative to the production in static flasks (Table 1) and production was more consistent than in shaken flasks. Similar results were obtained in bioreactors in both labs (i.e. at two independent locations) with reactors varying in size from 0.5 to 10 L scale. This improvement in scopularide A production in agitated conditions suggested that adequate air supply was important for scopularide A production, as was confirmed with an oxygen-limited bioreactor (10 mg scopularide A L^-1^, 0.7 mg [g biomass]^-1^, Table 1) in which scopularide A production was comparable to that observed in the static flasks.

**Table 1:** **Biomass and maximal scopularide A production by *S. brevicaulis *** **LF580 in bioreactor cultivations**

**Condition**	**Glucose (g L**^**-1**^**)**	**C/N (g g**^**-1**^**)**	**Biomass (g L**^**-1**^**)**	**Scopularide A (mg L**^**-1**^**)**^**†**^	**Time (h)**
Static flask - YMP [8]	10	10		7	~336
O_2_-limited batch-YMP^††^	11	10	11	10	90
Batch – YMP	11	10	11 ± 1	29 ± 3	90
N-limited batch-YMP	21	15	12 ± 1	8	74
Batch – YMP	20	11	15 ± 2	58 ± 4	90
Batch or fed-batch-YMP	44	10	25 ± 1	142 ± 20	167
Batch or fed-batch-YMP	61	10	33 ± 1	202 ± 58	165
Batch – CSS	17	7	12	44	90
Batch – defined, glucose	17	9	12	41	87
Batch – defined, glucose	40	9	24 ± 6	44 ± 7	100
Batch – defined, xylose	40	9	25 ± 1	54 ± 2	100

*S. brevicaulis* LF580 was grown in YMP, CSS or defined medium (and 30 g Sea Salt L^-1^) in batch or fed-batch culture at 28°C. Scopularide A was extracted from the mycelia at the times indicated. C/N values are estimated, as indicated in the methods. Average values are given, with ± sem for scopularide A and biomass, for cultivations which were carried out in replicate (n = 2 to 5). Published data from static flasks [8] is provided for comparison.

**Figure 1:**
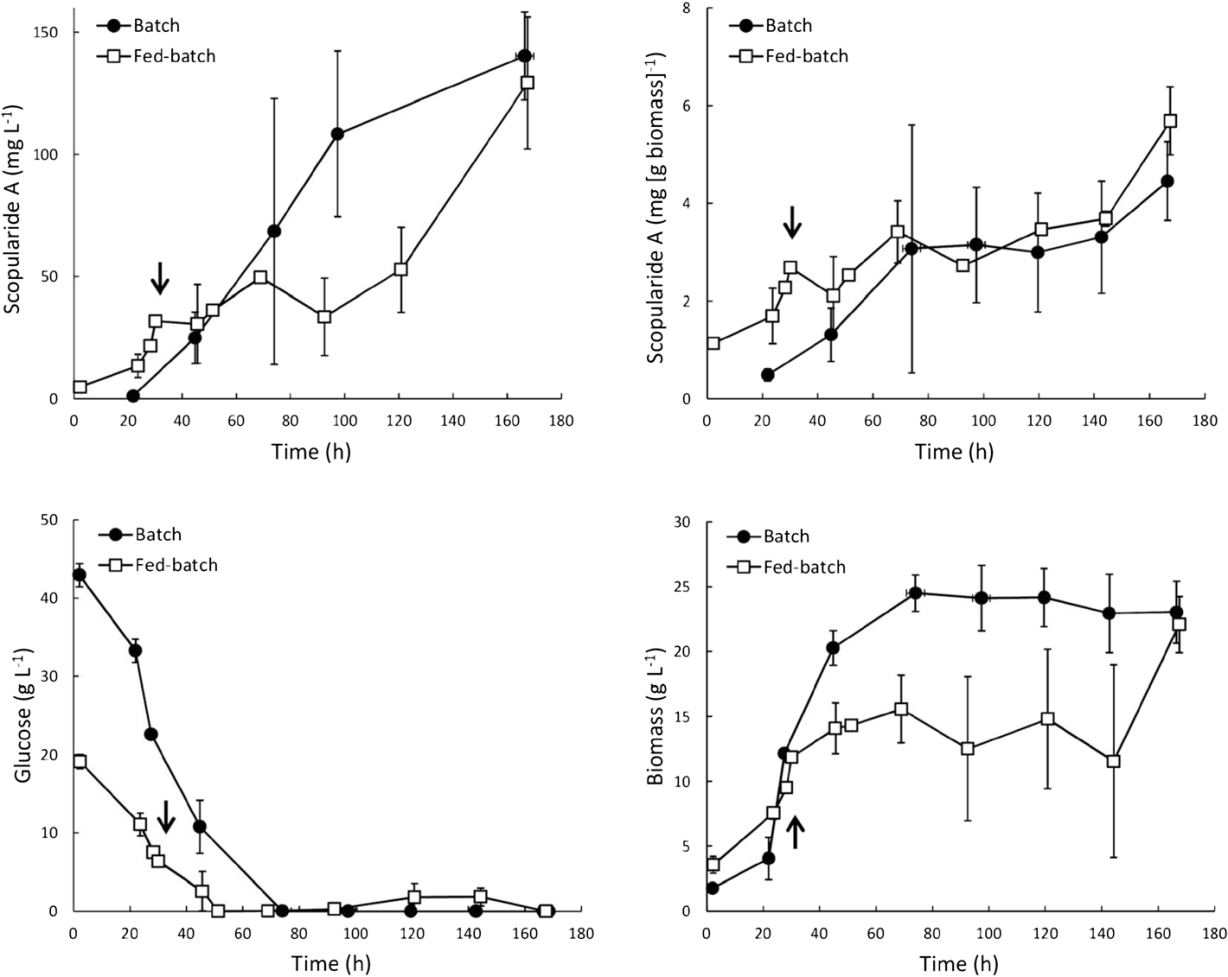
**Comparison of batch (solid circle) and fed-batch (open square) cultures of *S. brevicaulis *LF580.** Cultures were provided a final total of ~44 g L^-1^ glucose and were grown in YMP medium supplemented with (NH_4_)_2_SO_4_ (Table 1) and 30 g L^-1^ marine salt, as described in the methods. Cultures were agitated at 400-600 rpm to maintain DOT ≥ 30%. Arrows indicate the time at which medium started to be fed to the fed-batch cultures. Error bars represent ± sem for n = 3 (batch) and n = 2 (fed-batch) cultures.

^†^Determined from total dry biomass, volume of culture remaining and specific scopularide A content of the biomass (see Figure 1).

^††^O_2_-limited culture agitated at 300 rpm, with 0.6 vvm air.

The medium supported a maximum specific growth rate of 0.25 ± 0.01 h^-1^ and the production of 11.3 ± 1 g biomass L^-1^ (yield ~0.83 g biomass [g carbohydrate]^-1^, assuming that malt extract contained 63% available carbohydrate).

### Nitrogen-limitation limited scopularide A production

Scopularide A was primarily retained in the hyphae, with only 7 ± 1% of the total being extractable from the culture supernatant at the end of various cultivations. Scopularide A production might therefore be improved by increasing the amount of biomass produced, provided that sufficient oxygen is available, as in STRs.

When *S. brevicaulis* LF580 was grown in YMP medium with 20 g glucose L^-1^ without addition of (NH_4_)_2_SO_4_ scopularide A production was reduced to 8.1 mg L^-1^ (Table 1, 0.62 mg scopularide A [g biomass]^-1^). The mycelia grew exponentially at μ = 0.24 h^-1^ during the first 14 h, but then entered a prolonged deceleration phase, even though more than 14 g L^-1^ glucose still remained. YMP is a weak complex medium which contains approximately 0.76 g L^-1^ amino nitrogen, which is not necessarily all available to an organism, depending on its ability to hydrolyse all peptide bonds. When additional nitrogen (0.75 g (NH_4_)_2_SO_4_ L^-1^) was added to the culture at 47 h, CO_2_ production immediately increased and the remaining glucose was rapidly consumed, indicating that the culture had been nitrogen limited. Nitrogen limitation would be expected to reduce scopularide production, since nitrogen needed for biomass production would no longer be available for synthesis of non-essential metabolites and nitrogen incorporated into scopularides could be recycled for more important cellular processes. Addition of 1 g L^-1^ (NH_4_)_2_SO_4_ (0.21 g N) to YMP with 20 g L^-1^ glucose was sufficient to improve scopularide A production (58 mg L^-1^, 3.6 mg [g biomass]^-1^). We concluded that the carbon nitrogen ratio (C/N, g g^-1^) should be ≤ 11 for good scopularide A production.

### Production of scopularide A in complex and defined medium with high biomass concentrations

*S. brevicaulis* LF580 was grown in batch and/or fed-batch cultures with 20-60 g glucose L^-1^ in YMP medium supplemented with (NH_4_)_2_SO_4_. Fed-batch cultures were used to avoid oxygen limitation and glucose repression at high glucose concentrations, before it became apparent that scopularide A was being produced during growth, even in the presence of glucose (Figure 1). Oxygen limitation did not occur in any culture, since agitation speed was automatically adjusted to maintain dissolved oxygen at levels ≥ 30%. Specific scopularide A production was similar in batch and fed-batch cultivations, as was the final concentration of scopularide A (Figure 1) and amount of biomass produced (25 ± 2 and 25 ± 2 g biomass L^-1^, for batch and fed-batch cultures supplied with 44 g L^-1^ glucose, respectively, and 31 ± 1 and 36 g biomass L^-1^, when provided 61 g L^-1^ glucose).

**Figure 2:**
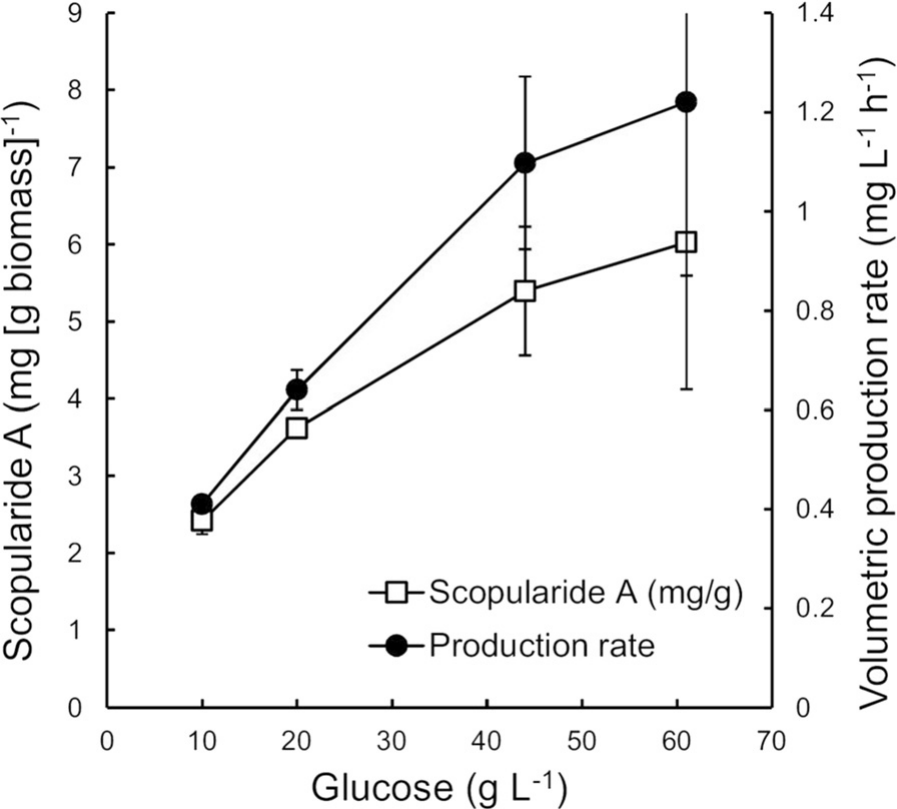
**Effect of glucose concentration on specific scopularide A production and the volumetric production rate.** Specific scopularide A production and volumetric production rate at time of maximal production (see Table 1). *S. brevicaulis* LF580 was grown in YMP (and 30 g Sea Salt L^-1^) with 11 to 61 g glucose L^-1^ in batch or fed-batch culture at 28°C. Scopularide A was extracted from the mycelia. Error bars are ± sem for n = 4, 2, 5 or 3 independent cultivations with ~10, 20, 40 or 60 g glucose L^-1^, respectively. Where not visible, error bars were smaller than the symbol.

The supply of glucose in fed-batch cultures did limit biomass production in the ~44 g L^-1^ glucose cultures (Figure 1), resulting in generally higher biomass and scopularide A concentrations in batch than in fed-batch cultures between 40 and 140 h. Since some scopularide A was also produced during the stationary phase of batch cultures, volumetric production rates were similar in both (e.g. 1.1 ± 0.3 and 1.0 ± 0.1 mg L^-1^ h^-1^, in batch and fed-batch cultures with ~44 g L^-1^ glucose, respectively). The slow initial biomass production in fed-batch cultures could be improved with a more optimal feeding profile, but no optimisation was undertaken, since fed-batch did not provide clear benefits. Since results were similar in batch and fed-batch cultures, final production data from the two methods have been combined (Table 1, Figure 2).

Both biomass and scopularide A production increased with increasing glucose provision (Table 1). Up to 202 ± 58 mg L^-1^ scopularide A were produced in cultures with 61 g L^-1^ glucose (Table 1), which was 29-fold higher than produced in the static flask cultures of Yu et al. [8]. The volumetric production rate was increased from 0.4 mg L^-1^  h^-1^ with 11 g L^-1^ glucose to 1.2 ± 0.3 mg L^-1^ h^-1^ with 61 g L^-1^ glucose (Figure 2).

Surprisingly, specific scopularide A production (mg [g biomass]^-1^) also increased with increasing glucose provision (Figure 2). Specific scopularide A production increased from 2.4 mg g^-1^ with 10 g glucose L^-1^ to 6.0 ± 1.9 mg g^-1^ with 60 g glucose L^-1^. Thus, even though there was greater variation in the amount of scopularide A produced in replicate cultures at high biomass concentrations than at low, volumetric production was improved more than expected by the 3-fold increase in biomass production.

Maximum specific growth rate (0.25 ± 0.01 h^-1^) was not affected by increasing the glucose concentration, but the yield of biomass on glucose and malt extract decreased, since yeast extract and soy peptone provided carbon as well as nitrogen for growth, but were added at a constant 3 and 5 g L^-1^, respectively, rather than in proportion to the glucose concentration, with additional nitrogen supplied as (NH_4_)_2_SO_4_.

Scopularide A was also produced in chemically defined medium, but at lower concentrations than in the corresponding cultures provided complex medium (Table 1). The specific growth rate of *S. brevicaulis* LF580 was reduced to 0.17 ± 0.03 h^-1^ in defined medium with glucose as carbon source, and 0.14 h^-1^ with xylose as carbon source, whereas biomass production (Table 1) and yield of biomass on carbohydrate were similar. *S. brevicaulis* LF580 did not grow on lactose (data not shown). The high biomass (40 g L^-1^ glucose) cultures were harvested after 100 h cultivation, after which time only small increases in scopularide A production had occurred in complex medium. This may have been too early for the slower growing defined medium cultures. However, most of the scopularide A in these cultures (47-50 mg L^-1^) was actually produced within the first 48 h and therefore we did not expect further production.

Similar amounts of scopularide A were produced on glucose or xylose, a less repressive carbon source, in defined medium (Table 1, ~2.3 mg [g biomass]^-1^). Scopularide A was produced in the presence of glucose concentrations up to 51 g L^-1^ in complex medium and its production did not appear to be repressed by glucose. Scopularide A may be considered a secondary metabolite in that it is not part of central metabolic pathways. However, unlike many secondary metabolites [15], scopularide A was produced during growth (Figure 1). Indeed, other peptide-derived fungal metabolites such as penicillin and cephalosporin are also produced during growth when carbon sources other than glucose are supplied [16], or when glucose supply is limited.

### Alternative nitrogen sources for scopularide A production

Yeast extract and soy peptone are relatively expensive sources of nitrogen, but could be replaced with corn steep solids (CSS). Similar amounts of scopularide A (3.6 mg [g biomass]^-1^ with 17 g L^-1^ glucose) were produced with CSS as the N-source, as in YMP. Each of these substrates provides short peptides and amino acids which generally support better growth than inorganic nitrogen sources by reducing the need to synthesise amino acids *de novo*. Trace elements, vitamins and some lipids are also provided. However, as sources of amino acids, they may also provide precursors (i.e. phyenylalanine, alanine, leucine, valine and glycine) for the scopularides. Yeast extract and soy peptone contain relatively higher amounts of all precursor amino acids than CSS, with the exception of alanine. The similar production of scopularides in CSS as in YMP suggests that amino acids from these sources were not serving as direct precursors for scopularide A production. More biomass was produced in YMP (15 ± 2 g L^-1^) than in CSS (12 g L^-1^), and thus volumetric scopularide A production was higher in YMP than in CSS (Table 1), which may indicate that the nitrogen in CSS was less accessible for growth than the peptides in yeast extract and soy peptone. None-the-less, Scopularide A can be produced in medium in which nitrogen is provided as amino acids from various complex sources or as ammonium, as in defined medium.

## Conclusions

Scopularide A production has been transferred from a non-agitated to a stirred tank bioreactor environment with approximately 6-fold increase in specific and 29-fold increase in volumetric production. Production of scopularide A in STRs demonstrates that marine fungal compounds can be suitable for scalable production, even with the native production organism, and similar strategies should be applied to enhance production of other recently discovered cyclic peptides which have only been produced in flask culture (e.g. [17-20]).

## Methods

### Strain

*Scopulariopsis brevicaulis* LF580 was obtained from the culture collection of the Kiel Center for marine natural products at GEOMAR, Helmholtz Centre for Ocean Research Kiel. Stock cultures were maintained as conidia suspended in 20% v/v glycerol, 0.8% w/v NaCl with ~0.025% v/v Tween 20 at -80°C. Spores were generated from cultures growing on agar-solidified YMP medium containing 30 g L^-1^ Sea Salt (Tropic Marin®, Germany).

### Media

Yeast Malt Peptone (YMP, containing 3 g yeast extract L^-1^, 3 g malt extract L^-1^ and 5 g soy peptone L^-1^) medium, modified from a yeast medium described by Wickerham [21] was used as the basic growth medium for *S. brevicaulis*. Glucose (10 - 66 g L^-1^) was provided as the primary carbon source. For agar solidified medium, YMP contained 10 g glucose L^-1^ and 15 g agar L^-1^. Yeast extract and soy peptone were replaced with corn steep solids (CSS, 8 g L^-1^) to assess the effect of an alternative peptide source. Media for high cell density cultures were supplemented with (NH_4_)_2_SO_4_ (1 to 9 g L^-1^) in proportion to the additional glucose provided, to ensure that cultures would not become N-limited.

The defined medium of Vogel [22], with 3.3 g L^-1^ (NH_4_)_2_SO_4_ substituted for NH_4_NO_3_ and glucose, xylose or lactose as the carbon source, was used to assess growth and scopularide production in defined medium. Supplementation of defined medium with CSS (5 g L^-1^) was also tested.

The composition of feed for fed-batch cultures is described in Table 2.

**Table 2:** Composition of the feed and the feeding rates used for fed-batch cultures

**Reactor**	**Feed composition**				
	**Glucose (g L**^**-1**^**)**	**(NH**_**4**_**)**_**2**_**SO**_**4 **_**(g L**^**-1**^**)**	Sea salt* (g L^**-1**^**)**	**Batch volume (L)**	**Feed rate (mL h**^**-1**^**)**
Qplus	100	21	30	0.75	2.4
CT5-2	220	50	30	4.5	5.5
C10-3	260	39	30	5	4.2

*S. brevicaulis* LF580 was grown in fed-batch cultures in 1 (Qplus), 5 (CT5-2) and 10 (C10-3) L bioreactors.

*Tropic Marin®.

Media for pre-cultures contained 4 g agar L^-1^ to facilitate filamentous growth. All media also contained 30 g Tropic Marin® Sea Salt L^-1^.

For determination of approximate C/N ratios, malt extract was assumed to contain 63% carbohydrate [23] and 1.1% total N [24], yeast extract to contain 9.8% total N [24], soy peptone 8.7% total N [24] and CSS 7.7% total N (based on information from Cerestar). Peptides (amino acids) were assumed to contain 47% carbon and CSS to also contain 5% carbohydrate (based on information from Cerestar).

### Cultural conditions

To assess scopularide production in shaken flasks, flasks (20 to 2000 mL, containing 10 to 1000 mL medium) were inoculated with conidial suspensions and incubated at 28°C, 120 rpm, for up to 170 h.

Pre-cultures for bioreactors were grown in 250 to 1000 mL Erlenmeyer flasks containing 20% medium on a volume basis, at 30°C, 200 rpm. Flasks were inoculated with conidial suspensions to give final concentrations of 3.4 × 10^5^ conidia mL^-1^. Pre-cultures were allowed to grow for 48 h (~19 g biomass L^-1^). Fermenters were inoculated with 10% final volume.

*S. brevicaulis* was grown in 500 mL (Multifors, max working volume 500 mL, Infors HT, Switzerland), 1 L (Biostat Qplus, max working volume 1.0 L, Sartorius AG, Germany), 5 L (Sartorius CT5-2, max working volume 5 L, Germany) and 10 L (Sartorius Biostat C10-3, max working volumes 10 L, Germany) bioreactors. Bioreactors were maintained at 28°C, with 700-800 (Multifors), 500 to 1200 (Biostat Qplus), 400 to 600 (CT5-2), or 400 to 800 (Biostat C10-3) rpm agitation and 0.5 (1-10 L cultures) or 1.7 (0.5 L cultures) volume gas (volume culture)^-1^ min^-1^ (vvm). Agitation in 1 to 10 L bioreactors was adjusted automatically to maintain the dissolved oxygen tension at or above 30%. The oxygen-limited culture was carried out in the Multifors bioreactor with 300 rpm agitation and 0.6 vvm aeration. Culture pH was kept constant at pH 7.0 by the addition of sterile 1 M KOH or 1 M H_3_PO_4_. Polypropylene glycol (1:1 mixture of M_n_ ~1000 and M_n_ ~2000, [25]) was added to control foam production.

Fed-batch cultures which were fed to a final concentration of ~44 g glucose L^-1^ were inoculated as batch cultures with 20 g L^-1^ glucose. Feeding was initiated at approximately 30 h, when glucose concentrations were < 9 g L^-1^. Fed-batch cultures which were fed to a final concentration of ~61 g glucose L^-1^ were inoculated as batch cultures with 40 g L^-1^ glucose, since no oxygen-limitation had occurred in the 40 g L^-1^ batch cultures. Feeding was initiated at approximately 47 h. Glucose was fed at rates which provided ~0.24 g L^-1^ h^-1^. The feed rate and concentration of glucose and ammonium in the feed (Table 2) were determined by the volume of the bioreactor and the capacity of the feed pumps associated with each bioreactor.

Samples were removed at intervals and mycelium was separated from the supernatant by centrifugation (3500 rpm 15 min, Eppendorf AG, centrifuge 5810 R, Germany). Mycelia were washed twice in water by centrifugation and freeze-dried (Christ, Freeze Drier, alpha 1-4 LD plus, Germany) to determine the biomass dry weight. Freeze-dried biomass was used for compound extraction.

Maximum specific growth rates were calculated from CO_2_ evolution rates. CO_2_ in the outlet gas was measured continuously in an Omnistar 16 quadrupole mass spectrometer (Balzers AG, Liechtenstein) calibrated with 3% CO_2_ in Ar, a Prima Pro Process mass spectrometer (Thermo Scientific, UK) calibrated with 3% CO_2_ in Ar, 5% CO_2_ with 0.99% Ar and 15% O_2_ in N_2_, 20% O_2_ plus 20% Ar in N_2_, and 0.04% ethanol in N_2_, or with a photoacoustic IR gas analyser (Innova-1313/LumaSense, United States) with air as reference.

### Chemical analyses

Scopularide A was extracted from mycelia or culture supernatant with ethyl acetate. Freeze-dried mycelia (10-50 mg) were fragmented in the presence of 1.8 mL ethyl acetate and 2 steel balls in a MM301 ball mill (2 × 3 minutes at 20 1/S, Retsch GmbH, Germany). Samples were centrifuged 10 min at 10000 rpm (Eppendorf AG centrifuge 5430) and the ethyl acetate phase transferred to a clean microfuge tube. Alternatively, scopularide A was extracted from 20 mL culture supernatant, from which mycelia had been removed by centrifugation, by vortexing with 20 mL ethyl acetate. Phases were separated by centrifugation (3500 rpm 15 min, Eppendorf AG, centrifuge 5810 R) and the ethyl acetate layer was retained. Ethyl acetate was evaporated under nitrogen and the solids re-dissolved in 200 μL HPLC grade methanol.

Ethyl acetate extracts were analysed by UPLC (ACQUITY UPLC, Waters) using a C18 UPLC column (1.7 μm, 2.1 mm × 100 mm, Waters; solvent isocratic (A) 70% (v/v) acetonitrile, (B) 5% (v/v) formic acid in H_2_O), and detected with a UV detector.

The concentration of D-glucose in culture supernatant was determined by HPLC using a Fast Acid Analysis Column (100 mm × 7.8 mm, BioRad Laboratories, Hercules, CA) linked to an Aminex HPX-87H organic acid analysis column (300 mm × 7.8 mm, 55°C, BioRad Laboratories) with 2.5 mM H_2_SO_4_ as eluant and a flow rate of 0.5 mL min^-1^. Peaks were detected using a Waters 410 differential refractometer. The concentration of ammonium was measured using a Potentiometer, Lab in the bag P (C-CIT.AG, Switzerland).

## Competing interests

The authors declare that they have no competing interests.

## Authors’ contributions

This study was conceived by MGW and AT. AT carried out cultivations and extractions at VTT. AL and AK carried out cultivations and extractions from flasks and bioreactors with 10 g L^-1^ glucose at GEOMAR. AT and MGW wrote the manuscript. All authors read and approved the final manuscript.

## References

[B1] Kobayashi J Ishibashi M Bioactive metabolites of symbiotic marine microorganisms Chem Rev 1993 93 1753 1769

[B2] Proksch P Edrada RA Ebel R Drugs from the seas – current status and microbiological implications Appl Microbiol Biotechnol 2002 59 125 143 12111137 10.1007/s00253-002-1006-8

[B3] Bhadury P Mohammad BT Wright PC The current status of natural products from marine fungi and their potential as anti-infective agents J Ind Microbiol Biotechnol 2006 33 325 337 16429315 10.1007/s10295-005-0070-3

[B4] Tarman K Lindequist U Wende K Porzel A Arnold N Wedssjohann LA Isolation of a new natural product and cytotoxic and antimicrobial activities of extracts from fungi of Indonesian marine habitats Mar Drugs 2011 9 294 306 21556160 10.3390/md9030294 PMC3083651

[B5] Le Ker C Petit K-E Biard J-F Fleurence J Search for hydrophilic marine fungal metabolites: a rational approach for their production and extraction in a bioactivity screening context Mar Drugs 2011 9 82 97 21339948 10.3390/md9010082 PMC3039472

[B6] Cai M Zhou X Lu J Fan W Niu C Zhou J Sun X Kang L Zhang Y Enhancing aspergiolide A production from a shear-sensitive and easy-foaming marine-derived filamenous fungus *Aspergillus glaucus* by oxygen carrier addition and impeller combination in a bioreactor Bioresour Technol 2011 102 3584 3586 21074418 10.1016/j.biortech.2010.10.052

[B7] Bringmann G Gulder TAM Lang G Schmitt S Stöhr R Wiese J Nagel K Imhoff JF Large-scale biotechnological production of the antileukemic marine natural product sorbicillactone A Mar Drugs 2007 5 23 30 18463724 10.3390/md502023 PMC2365691

[B8] Yu Z Lang G Kajahn I Schmaljohann R Imhoff JF Scopularides A and B, cyclodepsipeptides from a marne sponge-derived fungus, *Scopulariopsis brevicaulis* J Nat Prod 2008 71 1052 1054 18412398 10.1021/np070580e

[B9] Andrewes P Cullen WR Feldmann J Koch I Polishchuk E Reimer KJ The production of methylated organoantimony compounds by *Scopulariopsis brevicaulis* Appl Organometal Chem 1998 12 827 842

[B10] Anbu P Gopinath SCB Hilda A Lakshmipriya T Annadurai G Optimization of extracellular keratinase production by poultry farm isolate *Scopulariopsis brevicaulis* Bioresour Technol 2007 98 1298 1303 16884905 10.1016/j.biortech.2006.05.047

[B11] Khalaf SA El-Sayed ASA l-Methioninase production by filamentous fungi: i-screening and optimization under submerged conditions Curr Microbiol 2009 58 219 226 19048340 10.1007/s00284-008-9311-9

[B12] Mudau MM Setati ME Screening and identification of endomannanase-producing microfungi from hypersaline environments Curr Microbiol 2006 52 477 481 16732459 10.1007/s00284-005-0439-6

[B13] Yoder JA Benoit JB Zettler LW Effects of salt and temperature on the growth rate of a tick-associated fungus, *Scopulariopsis brevicaulis* bainier (Deuteromycota) Internat J Acarol 2003 29 265 269

[B14] Ding B Yin Y Zhang F Li Z Recovery and phylogenetic diversity of culturable fungi associated with marine sponges *Clathrina luteoculcitella* and *Holoxea* sp. in the South China Sea Mar Biotechnol 2011 13 713 721 21088979 10.1007/s10126-010-9333-8

[B15] Martin JF Demain AL Control of antibiotic biosynthesis Microbiol Rev 1980 44 230 251 6991900 10.1128/mr.44.2.230-251.1980 PMC373178

[B16] Revilla G López-Nieto MJ Luengo JM Martín JF Carbon catabolite repression of penicillin biosynthesis by *Penicillium chrysogenum* J Antibiot (Tokyo) 1984 37 781 789 6432764 10.7164/antibiotics.37.781

[B17] Liu S Shen Y A new cyclic peptide from the marine fungal strain *Aspergillus* sp. AF119 Chem Nat Compd 2011 47 786 788

[B18] Gulder TAM Hong H Correa J Egereva E Wiese J Imhoff JF Gross H Isolation, structure elucidation and total synthesis of lajollamide A from the marine fungus *Asteromyces cruciatus* Mar Drugs 2012 10 2912 2935 23342379 10.3390/md10122912 PMC3528133

[B19] Pérez-Victoria I Martín J González-Menéndez V de Pedro N El Aouad N Ortiz-López FJ Tormo JR Platas G Vicente F Bills GF Genilloud O Goetz MA Reyes F Isolation and structural elucidation of cyclic tetrapeptides from *Onychocola sclerotic* J Nat Prod 2012 75 1210 1214 22694270 10.1021/np3000987

[B20] Silber J Ohlendorf B Labes A Näther C Imhoff JF Calcaripeptides A-C, cyclodepsipeptides from a *Calcarisporium* strain J Nat Prod 2013 76 1461 1467 23865792 10.1021/np400262t

[B21] Wickerham LJ Taxonomy of yeasts US Dept Agric Wash DC Techn Bull 1951 1029 1 56

[B22] Vogel HJ A convenient growth medium for *Neurospora* (Medium N) Microb Genet Bull 1956 243 112 119

[B23] Neogen malt extract http://www.neogen.com/Acumedia/pdf/ProdInfo/7341_PI.pdf

[B24] Bridson EY The Oxoid Manual 8th Edition 1998 Basingstoke: Oxoid Limited

[B25] Wiebe MG Robson GD Shuster J Trinci APJ Evolution of a recombinant (glucoamylase-producing) strain of *Fusarium venenatum* A3/5 in chemostat cultures Biotechnol Bioeng 2001 73 146 156 11255162 10.1002/bit.1046

